# Analysis for stress environment in the alveolar sac model

**DOI:** 10.4236/jbise.2013.69110

**Published:** 2013-09

**Authors:** Ramana M. Pidaparti, Matthew Burnette, Rebecca L. Heise, Angela Reynolds

**Affiliations:** 1 Department of Mechanical and Nuclear Engineering Virginia Commonwealth University, Richmond, USA; 2 Department of Biomedical Engineering Virginia Commonwealth University, Richmond, USA; 3 Department of Applied Mathematics Virginia Commonwealth University, Richmond, USA

**Keywords:** Alveolar Sac, Stress, Analysis, Modeling

## Abstract

Better understanding of alveolar mechanics is very important in order to avoid lung injuries for patients undergoing mechanical ventilation for treatment of respiratory problems. The objective of this study was to investigate the alveolar mechanics for two different alveolar sac models, one based on actual geometry and the other an idealized spherical geometry using coupled fluid-solid computational analysis. Both the models were analyzed through coupled fluid-solid analysis to estimate the parameters such as pressures/velocities and displacements/stresses under mechanical ventilation conditions. The results obtained from the fluid analysis indicate that both the alveolar geometries give similar results for pressures and velocities. However, the results obtained from coupled fluid-solid analysis indicate that the actual alveolar geometry results in smaller displacements in comparison to a spherical alveolar model. This trend is also true for stress/strain between the two models. The results presented indicate that alveolar geometry greatly affects the pressure/velocities as well as displacements and stresses/strains.

## 1. INTRODUCTION

The alveolar region of the lungs plays an important role in breathing through the process of gas exchange. It occurs between the alveolar membrane and the underlying capillaries. During mechanical ventilation, the distribution of forced air within lung parenchyma results in the overdistension of the alveolar wall leading to a cascade of other conditions. These conditions include volutrauma/barotrauma (extreme stress/strain), atelectrauma (repeated opening and closing of collapsed alveoli) and biotrauma. If the aforementioned conditions are increased multi-system organ failure (MSOF) can occur [1,2].

The human alveolus is extremely small with only a thin membrane separating the air from the pulmonary capillaries. The sheer number of alveoli makes up for their small size, giving a total gas exchange surface area of 143 m^2^ [3]. The alveoli are clustered together to form alveolar sacs, with various complex geometries. There are many types of cells found in the alveolar walls, including secretory and granular pneumocytes [4]. Lining the airway passages in the alveoli is surfactant, which reduces alveolar surface tension. The alveoli are supported by a network of collagen and elastin fibers, providing recoil and essentially making a netting for the alveoli [5,6]. Damage to the netting of collagen and elastin fibers can lead to large stress and deformation changes in the alveolar wall, causing further issues in the lungs [5,7]. This damage can be caused by numerous diseases such as pulmonary fibrosis, emphysema, COPD, or asthma [7]. Many of these diseases deteriorate the lung, especially in the region of the lower lung comprising of the alveoli. Alveolar compromise leads to acute respiratory distress syndrome (ARDS) in which the mechanical properties of the lung parenchyma such as lung compliance are decreased [4].

Computational models have been used in the past to better understand the internal stresses of the lungs. They have also been used to understand the changes that occur when a patient is on a ventilator. Numerous models have also examined the nature of the collagen and elastin fibers in their role of supporting the alveoli [8–11]. Very few models have tried to simulate an actual alveolus or alveolar sac, since the geometry is complex and the size is so small. Normally simple geometric approximations are used [11]. The exact nature of the airflow at the alveolar level is not understood completely even though various approximations have been made.

Several studies focused on the effects of mechanical ventilation on the diseased and healthy lungs at the tissue and cellular levels. Li *et al*. [12] studied the airflow analysis in the alveolar region showing the influence of geometry structure on the airflow field and pressure distributions. However, this study gives no information on the mechanical forces induced by the interaction between the air and alveolar wall. A 2-D fluid structure analysis study by Dailey *et al*. [13] investigated how tissue mechanical properties and breathing patterns influence deep-lung flow fields and particle dynamics. However, this did not include mechanical ventilation. Recently, Rausch and colleagues [14] investigated the local strain distribution by using a finite element simulation of X-ray tomographic microscopy scanned alveolar geometries. They were able to model the alveoli wall and attain strain distributions for single alveolar wall. They did not consider the mechanical ventilation boundary condition in their study.

In order to better understand single alveolar sac airway mechanics, a three-dimensional model of the actual alveolar sac model was developed in this study and investigated the stress/strain environment using a coupled fluid-solid analysis. In addition, an idealized spherical model is also developed and the results obtained are compared between the two alveolar sac models.

## 2. MATERIALS AND METHODS

**Figure 1:**
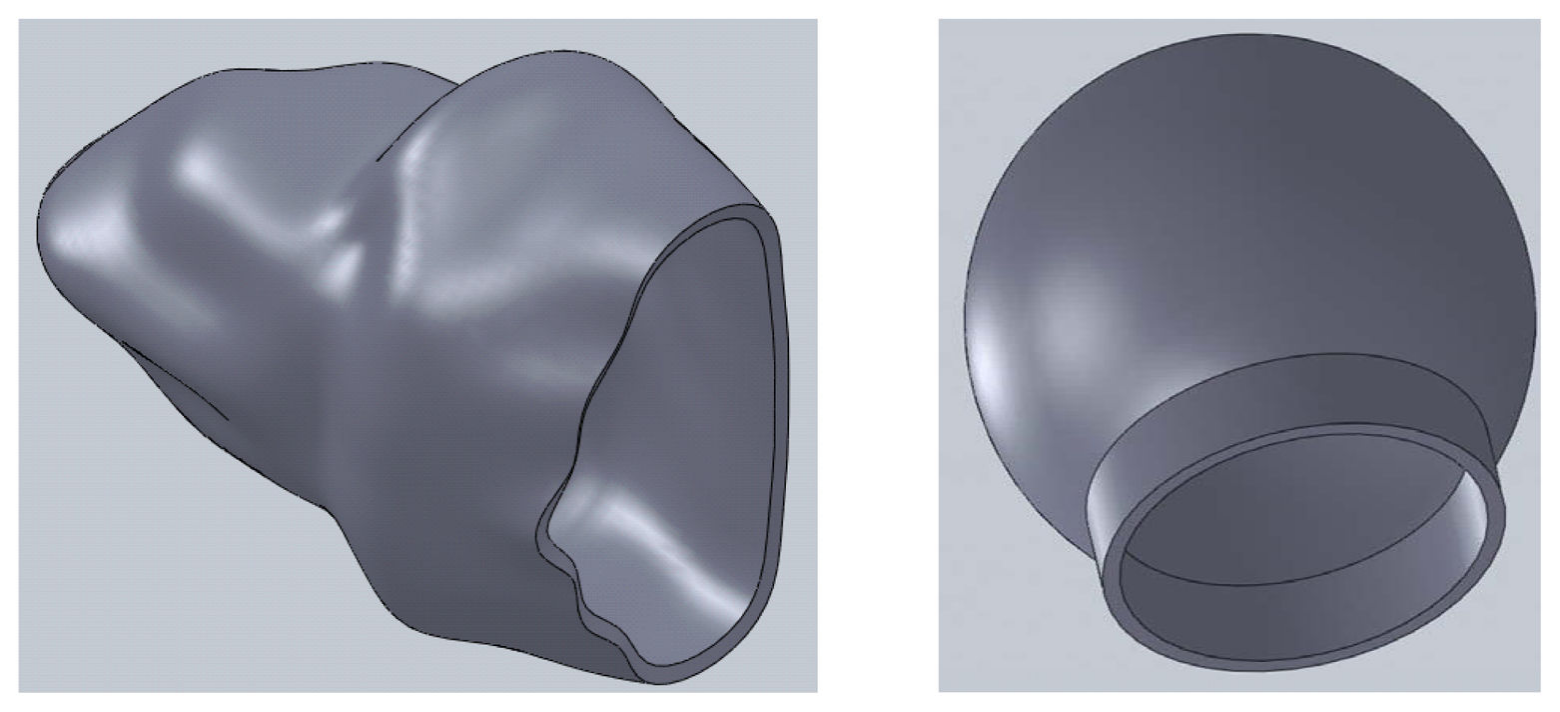
Alveolar Sac models: Actual (left) and Spherical (right).

Two alveolar sac models, one with actual geometry obtained from a scanning electron micrograph (SEM) of a typical alveolar sac from a rabbit lung from Bachofen and Schürch [15]. A second model representing a spherical sac was also created with the same inlet area and volume as the actual alveolar sac model. Both the geometries of the alveolar sac model in 3D model was created using Solid Works as shown in Figure 1. The use of a rabbit lung is justified in that the general shape of mammalian lung structures at the microscopic level is similar [7]. However, the thickness of the septal walls had to be changed to match human lungs [3], with an average thickness of 8 μm was used in the computer model [7]. The properties used in the model development are given in Table 1.

The computational model involves both fluid and solid domains of the alveolar sac. The solid domain is the alveolar sac itself, while the fluid domain is the air contained within the sac. The alveolar sac was considered as rigid (no deformation) in fluid analysis through computational fluid dynamics (CFD) from which the airway were obtained. However, from coupled fluid-solid analysis through fluid-structure interaction, the strains on alveolar sac were obtained by considering the alveolar sac tissue as physiologically compliant (able to deform). The transient interactions between airflow and sac tissue during mechanical ventilation were investigated by solving two coupled sets of governing equations with associated boundary conditions. The governing equations for airflow and airways are briefly described below.

### 2.1. Airflow Equations

The governing equations for transient airflow are the Navier-Stokes equations on a moving mesh with the assumption of incompressible flow. These equations govern the principles of mass and momentum conservation and are described below.

Conservation of mass:

(1) $$\frac{\rho_{g}}{\sqrt{g}}\frac{\partial }{\partial t}(\sqrt{g})+\rho_{g}\frac{\partial }{\partial x_{j}}\left(u_{j}-\frac{\partial \;{\tilde{x}}_{j}}{\partial t}\right)=0$$

Conservation of momentum:

(2) $$\frac{\rho_{g}}{\sqrt{g}}\frac{\partial }{\partial t}\left(\sqrt{g}u_{i}\right)+\rho_{g}\frac{\partial }{\partial x_{j}}\left[\left(u_{j}-\frac{\partial \;{\tilde{x}}_{j}}{\partial t}\right)u_{i}\right]=-\frac{\partial p}{\partial x_{j}}+\mu \frac{\partial^{2}u_{i}}{\partial x_{j}^{2}}$$

In these equations *x̃_j_* represents the moving mesh location, $\sqrt{g}$ is the metric tensor determinate of the transformation, *i.e.* the local computational control-volume size, *ρ_g_* is the fluid density, *p* is the fluid pressure, *μ* is the fluid viscosity, and *u* is the fluid velocity [1,2].

### 2.2. Alveolar Sac Wall Equations

The governing equations for the movement of the alveolar sac walls during inhalation and exhalation are the time-dependent structural equations described below using Einstein’s repeated index convention.

Equation of Motion:

(3) $$\frac{\partial \sigma_{ij}}{\partial x_{j}}+F_{i}=\rho \frac{\partial^{2}u_{i}}{\partial t^{2}}$$

Constitutive Relations:

(4) $$\sigma_{ij}=C_{\mathrm{ijkl}}\varepsilon_{kl}$$

In the equations above *σ* is the stress in each direction, *F* is the body force, *ρ* is the density, *u* is the displacement, *C* is the elasticity tensor, and *ε* is the strain in each direction [1,2].

### 2.3. Computational Simulations

The alveolar sac models generated from Solid Works software were imported into ANSYS Workbench, where FSI was conducted using ANSYS Mechanical (Version 12.1) and ANSYS CFX (Version 12.1). ANSYS Mechanical is a general finite element (FE) software program for structural modeling, and ANSYS CFX is a general purpose computational fluid dynamics (CFD) software program for modeling fluid flows. The individual models were coupled using a fluid-structure interaction (FSI) algorithm [2]. Analysis assumed the solid portion was compliant, *i.e.* able to move. The fluid model equations were solved first to obtain fluid pressures, which were then applied to the solid model. Displacements were solved for in the solid model equations, which were then applied to the fluid model. The fluid model equations are resolved using the structural displacements at the boundaries. The process iterates until a converged solution is found for each time step. The equations for each domain are described above.

### 2.4. Finite Element Meshes

**Figure 2:**
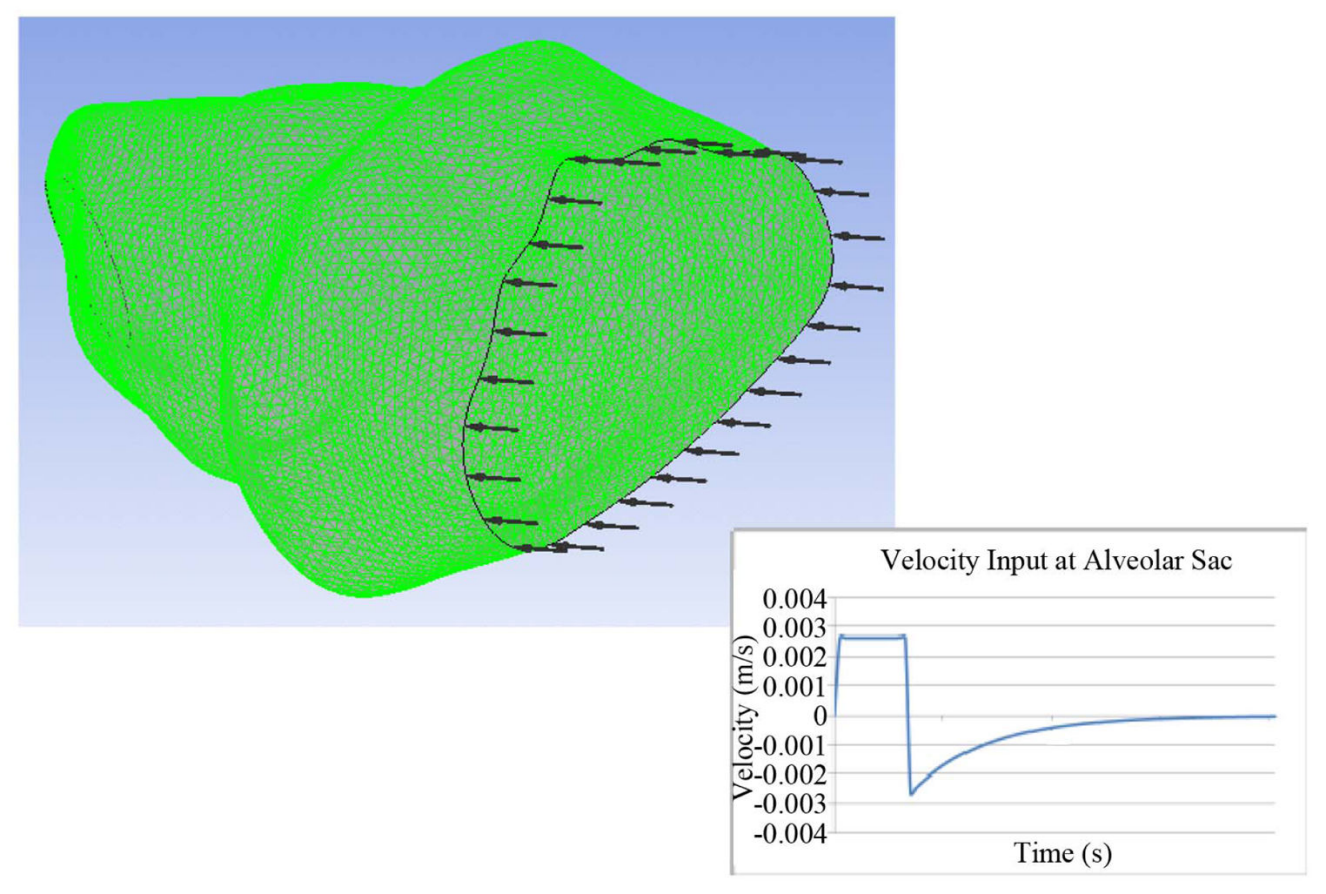
Finite element model (left) and mechanical ventilation waveform used in the analysis.

The fluid domain of the alveolar sac model was comprised of 1,364,551 tetrahedral elements with 238,688 nodes, and the solid domain had 135,129 tetrahedral elements with 222,762 nodes (see Figure 2). The fluid domain of the spherical model was comprised of 1,459,612 tetrahedral elements with 252,937 nodes, and the solid domain had 135,463 tetrahedral elements with 225,338 nodes. The inlets for both the fluid and solid domains were fixed, *i.e.* zero displacement. A no slip boundary condition was applied at the fluid-solid interface.

### 2.5. Tissue Parameters

For the alveolar sac model, the wall was assumed to be made of a homogeneous and linearly elastic material [1,2]. The wall has a density of 196 kg/m^3^, Young’s Modulus of 5 kPa, and a Poisson’s ratio of 0.4 [7]. The air was assumed to be an incompressible fluid at 25°C.

### 2.6. Mechanical Ventilation Parameters

The airflow rate during mechanical ventilation is assumed to be a constant 60 L/min for inhalation at the tracheal level. Inhalation occurs for 0.7 seconds to correspond to a tidal lung volume of 700 cm^3^ as shown in Figure 2. Passive exhalation then occurs, which is given by the equation:

(5) $$v(t)=-\frac{e^{\frac{t}{\tau }}}{A_{i}}$$

where *v* is the airflow velocity (m/s), *t* is time (s), *τ* is the time constant, and *A_i_* is the cross-sectional area of the inlet. Both the inhalation and exhalation are scaled to obtain the velocity in the alveolus assuming that the flow divides equally for 24 bifurcations. The waveform is based off of the waveform used by Pidaparti *et al*. [1,2]. This flow rate is applied as the inlet boundary condition of the fluid domain.

## 3. RESULTS AND DISCUSSION

For presenting analysis results, two times were selected as representable for all times, since the actual analysis was run for a total simulation time of 4.01 seconds, with time steps of 0.1 seconds, giving over 400 time steps.

### 3.1. Pressures and Velocities

**Figure 3:**
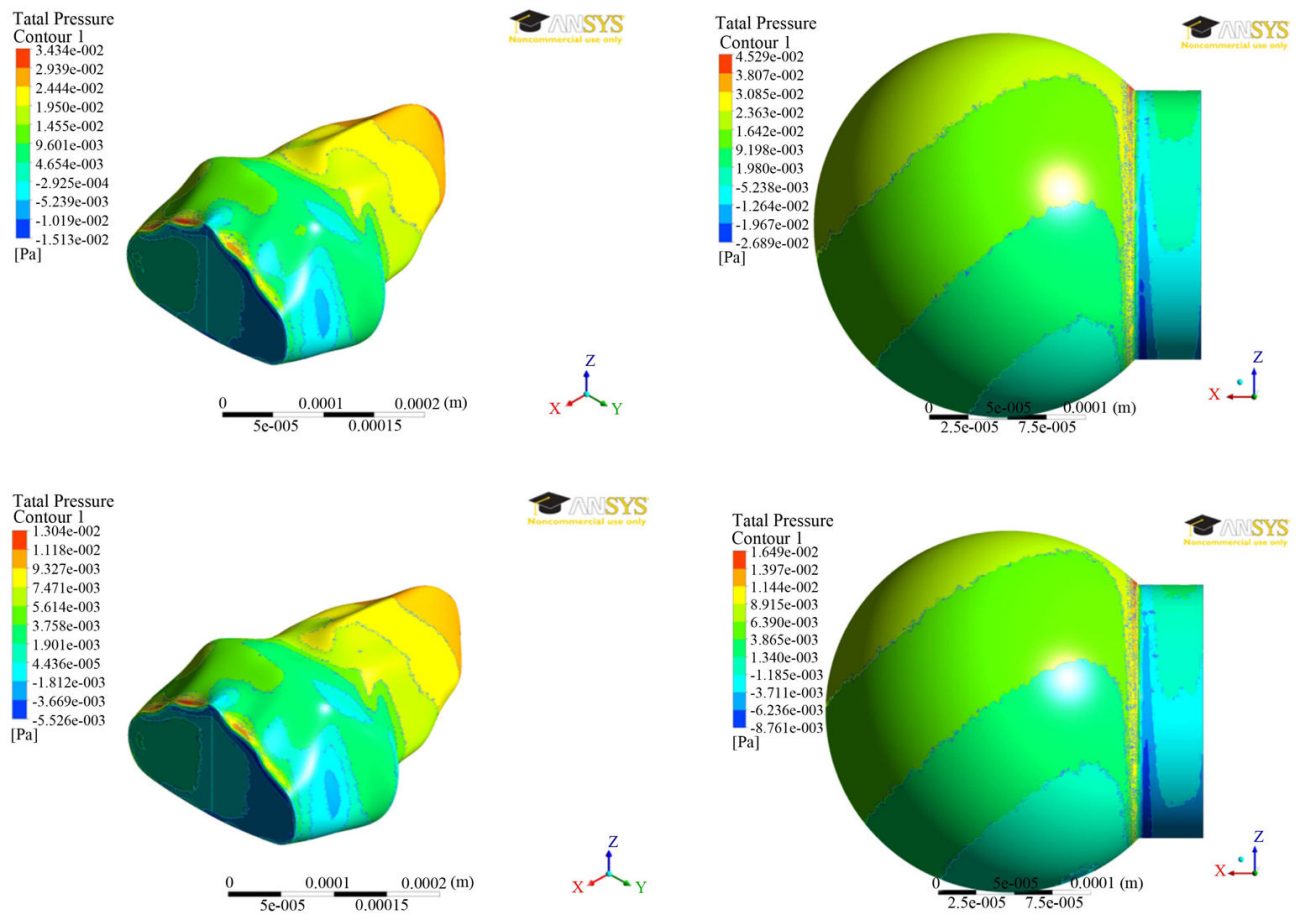
Pressure distributions at two different times (0.7 secs—top; 1.4 secs—bottom) between the two alveolar models.

The results of pressure distributions for both the models were presented in Figure 3 at two different times (0.7 and 1.4 secs). The results are similar at both the times. It can be seen from Figure 3 that the pressure was greatest near the inlet for inhalation and greatest near the far end from the inlet for exhalation. The exceptions to this were near the zero-displacement boundary at the inlet for the alveolus model and at the stress concentration (where the sphere meets the inlet) for the spherical model. Ignoring the higher pressures due to the exceptions above, the average pressures were approximately the same for the alveolar and spherical models, being around 15 MPa, and 7 MPa for the time steps of 0.7 seconds, and 1.4 seconds, respectively.

**Figure 4:**
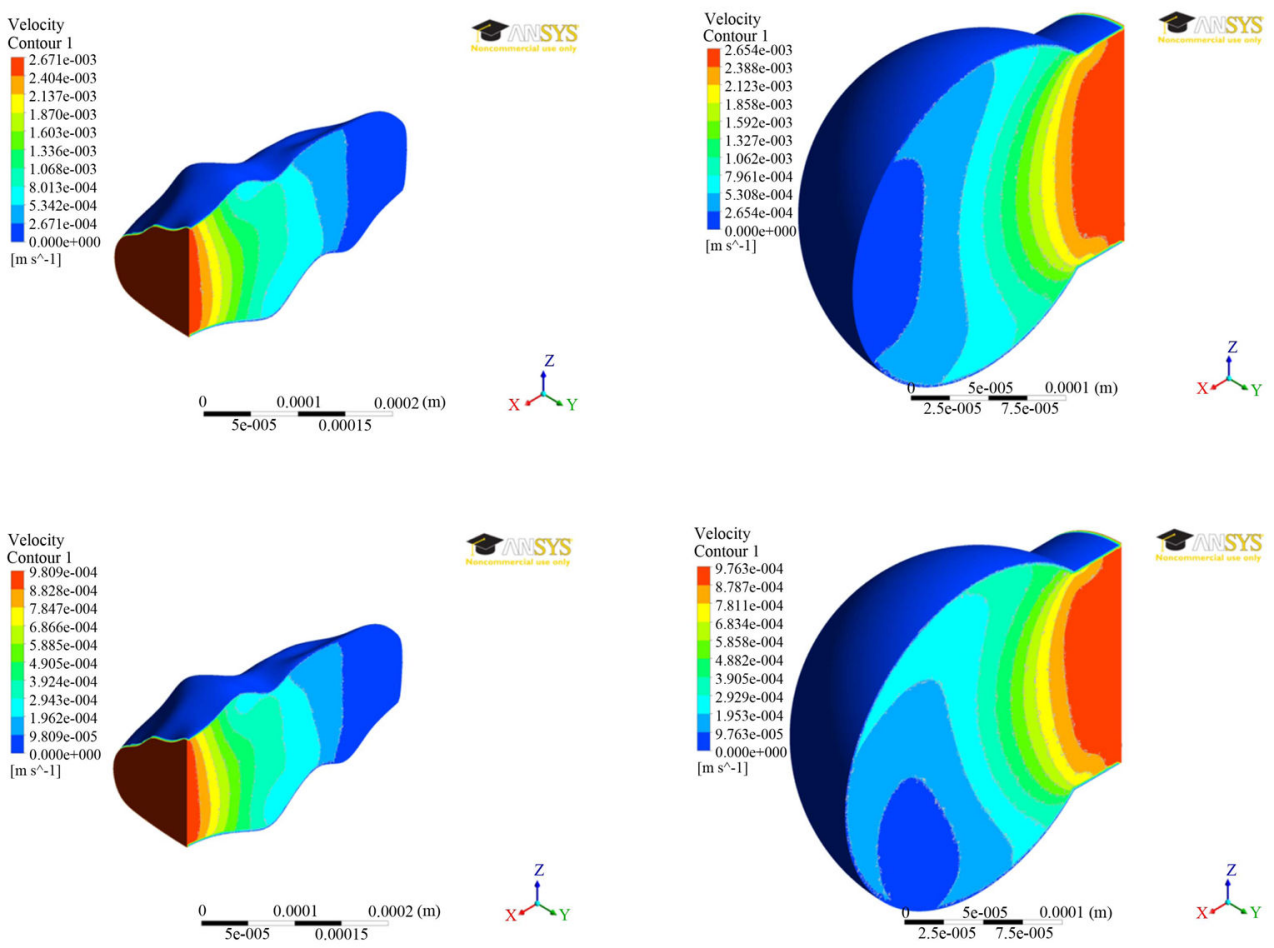
Velocity distributions at two different times (0.7 secs—top; 1.4 secs—bottom) between the two alveolar models.

The results of velocity distributions for both the models were presented in Figure 4 at two different times (0.7 and 1.4 secs). The outside surface has zero velocity due to the no-slip condition applied at the wall. It can be seen from Figure 4 that the velocity decreased with distance toward the inside of the model. The maximum velocities are comparable, being around 2.7 mm/s for time step of 0.7 seconds and around 0.98 mm/s for the time step 1.4 seconds.

The results from fluid analysis between the two fluid models are very similar, with no large differences in pressure or velocity, particularly in magnitude. Due to the greatly different geometries, the pressure and velocity waveforms have different appearances, but ignoring the changes in shape, the overall waveforms are similar.

### 3.2. Displacements/Stresses

**Figure 5:**
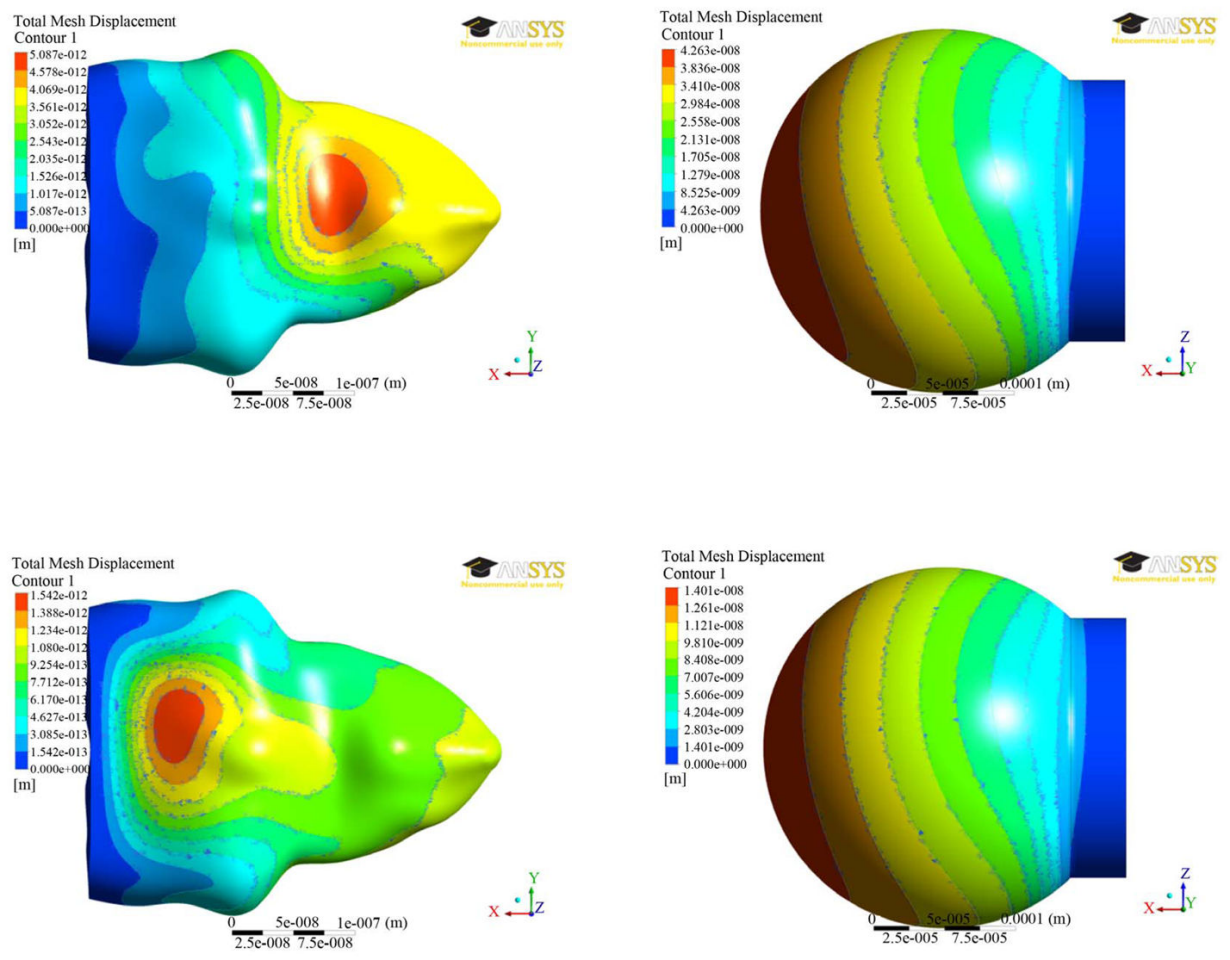
Displacement distributions at two different times (0.7 secs—top; 1.4 secs—bottom) between the two alveolar models.

The results of displacement distributions for both the models were presented in Figure 5 at two different times (0.7 and 1.4 secs). The results are similar at both the times. It can be seen from Figure 5 that the displacement contours for the spherical model do not change significantly. However, the maximum of 43 nm was found to be at 0.70 seconds and decreases to 14 nm at 1.4 seconds. For the alveolar model, the displacements are much smaller in magnitude and vary locations for maximum displacement. At 0.7 seconds, the greatest displacement is on a large concave area of the alveolus. At 1.4 seconds, a relatively flat area has the greatest displacement at a magnitude of 1.5 nm.

**Figure 6:**
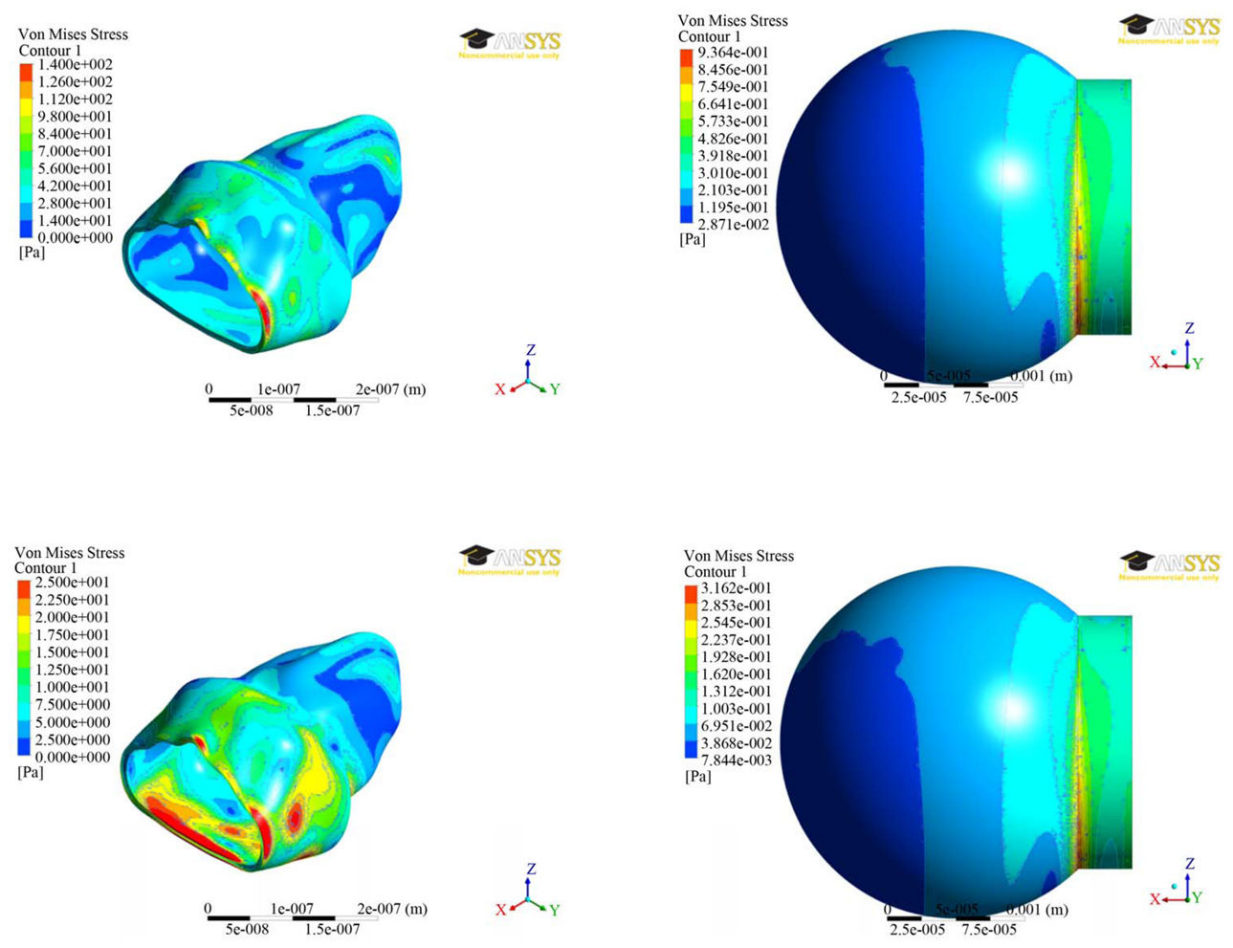
von-Mises stress distributions at two different times (0.7 secs—top; 1.4 secs—bottom) between the two alveolar models.

The results of von-Mises stress distributions for both the models were presented in Figure 6 at two different times (0.7 and 1.4 secs). The Von mises stress is a measure of the total stress at a location, taking into accounts both shear and normal stresses. For the spherical model, the stress did not vary much over the sphere, but large stress concentrations occurred where the sphere intersects the straight portion of the inlet. The maximum stress of 936 mPa was found at time step 0.7 seconds and a stress of 316 mPa at time step 1.4 seconds. The stress contours for the alveolus model varied greatly depending on whether inhalation or exhalation was occurring. Nevertheless, the greatest stresses were at the zero displacement boundary, as listed in Table 2. For the Figure 5, a user-defined scale was used to better highlight the variation in stress away from the zero displacement boundaries at the inlet. Ignoring the high stresses due to the zero displacement boundaries, the highest stresses occurred where there was a drastic change in the shape of the alveolus.

The alveolar model experienced smaller strains but much greater stresses than the spherical model. This is partially explained by the different geometries, but the fact that they are different by a few orders of magnitude is highly unusual. For the displacements, the results are about as expected, excluding the large magnitude variations. The largest displacements occur at different locations due to geometry variations. The sphere distributed the stress fairly evenly all around the sides, but the alveolus model did not due to the varied geometry. The various contours in the body created numerous areas of stress concentration.

Based on the coupled fluid-solid analysis results, the displacements and stresses are vastly different between the alveolar sac model and the spherical model. The difference in geometry and possibly the difference in entrance lengths to the two alveolus models caused small displacements and high stresses to occur in the alveolus model compared to the spherical model.

## 4. CONCLUSION

Two different alveolar sac models were developed, one based on actual geometry and the other an idealized spherical geometry. Both the models were analyzed through coupled fluid-solid analysis to estimate the alveolar mechanics parameters such as pressures/velocities and displacements/stresses. From the fluid analysis, results obtained indicate that both the alveolar geometries give similar results for pressures and velocities. However, the results obtained from coupled fluid-solid analysis indicate that the actual alveolar geometry results in smaller displacements in comparison to a spherical alveolar model. This trend is also true for stress/strain between the two models. The results presented indicate that alveolar geometry greatly affects the pressure/velocities as well as displacements and stresses/strains. More research is needed to further investigate how these models affect the actual strains set-up within the alveolar tissue, which is being pursued as a future work.

## Figures and Tables

**Table 1:** Alveolar model parameters.

Volume	8735202.83 μm^3^
Solid Volume	1593056.73 μm^3^
Fluid Volume	7142146.09 μm^3^
Length	335.14 μm
Inlet Area	22458.54 μm^2^

**Table 2:** Von mises stresses.

Time (sec)	Max Stress (Pa)	Min Stress (Pa)
0.7	201.4	2.213
1.4	59.50	0.2519
